# Prominent Eustachian Valve Mimicking Inferior Rim of Atrial Septum Causing Iatrogenic Inferior Vena Cava Type Sinus Venosus Atrial Septal Defect

**DOI:** 10.7759/cureus.15387

**Published:** 2021-06-02

**Authors:** Annie T Wang, Uoo Kim

**Affiliations:** 1 Anesthesiology and Perioperative Medicine, Loma Linda University Medical Center, Loma Linda, USA

**Keywords:** eustachian valve, transthoracic and transesophageal echocardiography, atrial septal defect, foramen ovale, septum primum, septum secundum, veno-arterial shunting, hypoxemia

## Abstract

The Eustachian valve is an embryologic remnant at the junction of the inferior vena cava (IVC) and right atrium (RA). While it typically does not have any pathologic significance, veno-arterial shunting can rarely occur in patients with prominent eustachian valves and atrial septal defects (ASD), causing cyanosis and hypoxemia despite normal pulmonary pressures. We present a case of a patient with iatrogenic residual sinus venosus IVC-type ASD secondary to a prominent Eustachian valve that was misinterpreted as the inferior rim of the atrial septum during initial ASD repair.

## Introduction

The Eustachian valve is an embryologic remnant that lies at the junction of the inferior vena cava (IVC) and inferior right atrium (RA). In fetal development, the valve directs incoming oxygenated blood towards the foramen ovale and away from the right atrium. The eustachian valve typically does not have any pathologic significance after the closure of the foramen ovale [1]. The embryologic remnant can present differently on echocardiogram, ranging in thickness, length, and shape. While the valve is usually benign, veno-arterial shunting is a very rare phenomenon that has been described in a few case reports [2-4]. It can occur in patients with prominent valves and atrial septal defects (ASD) without pulmonary hypertension or right ventricular outflow obstruction [2-4]. We report a case of a patient with iatrogenic residual IVC type sinus venosus ASD secondary to a prominent Eustachian valve that was misinterpreted as the inferior rim of the atrial septum during the initial ASD repair. Written informed consent was obtained from the patient.

## Case presentation

A 59-year-old morbidly obese female (body mass index of 36 kg/m^2^) with history of congenital ASD, which was initially diagnosed and surgically repaired at age 19, was admitted for cardiac evaluation due to several months of dyspnea at rest and with exertion, palpitations, fatigue, intermittent chest pain, and hypoxemia. Additional comorbidities included coronary artery disease with six prior myocardial infarctions (last myocardial infarction in 2010), atrial flutter, hypertension, and type 2 diabetes mellitus controlled with oral medications. Medications included metoprolol, lisinopril, amiodarone, dabigatran, aspirin, metformin, and atorvastatin. On physical examination, the patient had a grade 1/6 systolic ejection murmur heard in the left upper sternal border, a mildly loud P2, decreased bilateral breath sounds with O2 saturations 94% on 2L NC (88% on room air), bilateral clubbing of both hands, and 1+ pitting edema in bilateral lower extremities. Laboratory values were within normal limits, and chest X-ray demonstrated mild cardiac enlargement without edema.

**Figure 1:**
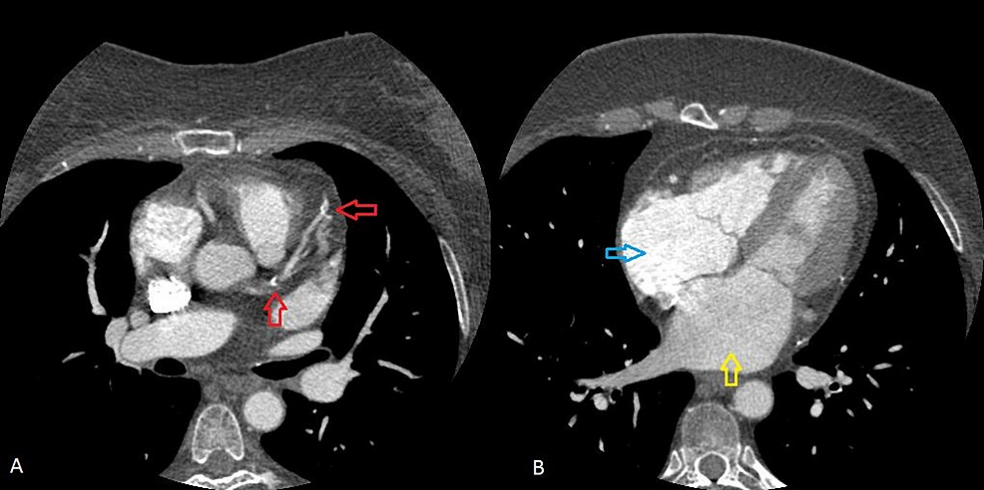
Cardiac CT scan. A: red arrows indicate coronary artery calcifications.  B: right and left atrial enlargement is seen (blue and yellow arrows, respectively).

Transthoracic echocardiogram demonstrated a residual ASD with right-to-left shunting. Bi-atrial enlargement was also noted, with a mild decrease in right ventricular systolic function. The left ventricle had a normal ejection fraction of 65% with normal wall motion. Pulmonary pressures were normal with a pulmonary arterial systolic pressure (PASP) of 25 mmHg. Right heart catheterization confirmed the residual ASD with right-to-left shunting of blood from the RA to LA at a ratio of 1.3:1. Pulmonary arterial pressures were within normal range. The cardiac pressures and saturation are listed in Table 1. Additionally, CT angiogram of the heart revealed moderate left atrial and severe right atrial enlargement and mild coronary artery calcification (Figure 1).

**Table 1:** Right heart catheterization pressures and oxygen saturation. LV: left ventricle; LA: left atrium; PV: pulmonary vein; MPA: main pulmonary artery; RA: right atrium; RV: right ventricle; SVC: superior vena cava; IVC: inferior vena cava.

	LV	LA	PV	MPA	RA	RV	SVC	IVC
Pressure (mmHg)	-	11	11	29/15	9	29/8	-	12
Oxygen saturation at FiO_2_ 60% (%)	91	95	96	80	82	78	73	73
Oxygen saturation at FiO_2_ 21% (%)	-	93	94	-	88	-	73	-

The patient subsequently underwent redo sternotomy with redo repair of the ASD with a bovine pericardial patch, takedown of the IVC to LA baffle, and ligation of the LA appendage. Intraoperative transesophageal echocardiogram (TEE) in the mid-esophageal bicaval and four-chamber view revealed a portion of the eustachian valve stitched to the inferior rim of the ASD, directing blood flow from the IVC towards the left atrium (LA) with right-to-left shunting (Video 1). A fenestration of 1 cm in the patch allowed communication between the IVC and RA. The left atrium was severely enlarged, with a massively dilated IVC, and mildly dilated right atrium with a severely dilated right ventricle. 

**Video 1:** Transesophageal echocardiogram prior to repair.

The eustachian valve of the IVC had been used for closure of the defect in the initial repair of the ASD, thought to be the inferior rim of the atrial septum which was completely absent. The large ASD appeared to be untouched. During the surgery, the eustachian valve was detached from the wall of the IVC, and the IVC was separated from the septum and the LA. IVC flow was restored to the RA. TEE confirmed absent residual shunting at the atrial level with the IVC flow directed to the RA, and all defects were closed (Video 2). The heart recovered with normal sinus rhythm with excellent hemodynamics. The patient was eventually discharged without any postoperative issues. She no longer required supplemental oxygen, and all prior symptoms resolved.

**Video 2:** Transesophageal echocardiogram post repair.

## Discussion

Persistent Eustachian valves can present with differences both anatomically and functionally. These may present with a wide range in shape, size, and location [1]. Incomplete regression of the Eustachian valve can appear as a thin, membranous flap or a thickened ridge at the IVC and right atrial junction [1]. It can also appear as a mobile mass within the RA, and large valves can be mistaken for tumors, thrombi, or vegetations in the RA [1]. Despite its persistence in adults, patients are typically asymptomatic and do not require any management. While complications are rare, one case report described a patient with a prominent Eustachian valve and ASD that likely contributed to a paradoxical brainstem embolism [5]. With such unpredictability in its appearance and function, a prominent Eustachian valve encroaching on the lower portion of the atrial septum can easily be misconstrued as the inferior rim of the ASD, which was what occurred in our patient.

Patients with uncomplicated ASDs with typical left-to-right shunting usually do not present with signs of cyanosis [3]. However, reversal in the direction of flow causing right-to-left shunting can result in cyanosis and hypoxemia. Typical mechanisms for this occurrence include increased right ventricular pressure from pulmonary arterial hypertension (Eisenmenger syndrome), right ventricular outflow obstruction from pulmonic stenosis, a large ASD resulting in equal biatrial pressures, increased venous return during termination of a Valsalva strain, and persistent left SVC syndrome [3,6]. However, our patient had normal pulmonary pressures without any evidence of right ventricular hypertrophy or valvular issues, which is atypical for right-to-left shunting. Our patient did not have any findings of persistent left SVC syndrome on TEE. Additionally, our patient’s symptoms were constant at rest and with exertion, so straining and/or Valsalva was unlikely the cause for hypoxemia. Hence, for our patient, the worsening hypoxemia with right-to-left shunting was most likely attributed to the Eustachian valve. Morishita described two patients who developed cyanosis with ASD and flow directed from the IVC to the LA, despite normal right ventricular pressures [2]. The cyanosis in these patients was attributed to persistent, large inferior vena caval Eustachian valves [2]. While few case reports have described similar veno-arterial shunting in patients with Eustachian valve and ASD [2-4,6-7], the cause was identified properly and surgically treated in a timely manner at a relatively young age. For our case, however, the Eustachian valve was misidentified incorrectly as the inferior rim of the ASD during the initial surgery. While the patient may not have had significant veno-arterial shunting prior to the initial surgery, the surgical manipulation of the valve could have consequently resulted in worsening right-to-left shunting from obstruction of flow from IVC to RA. Over time, the patient’s veno-arterial shunting likely worsened until the patient developed clinical signs and symptoms many years later. 

Distinguishing the Eustachian valve from other neighboring structures is critical. For our patient, misidentifying the Eustachian valve as a portion of the inferior atrial septum during the initial ASD repair resulted in significant iatrogenic right-to-left shunting, causing worsening hypoxemia. In this situation, IVC flow was directed preferentially across the ASD towards the left atria, resulting in a severely dilated LA. While there was a small fenestration that allowed communication between the IVC and RA, the LA was still overwhelmed with the flow across the right-to-left shunt. The IVC was also noted to be massively dilated, which could signify that the Eustachian valve was obstructing blood flow from the IVC to RA. With the close proximity of the Eustachian valve to the atrial septum, there have been few case reports describing the iatrogenic diversion of blood flow from the IVC to LA during ASD repair in patients with low-lying ASDs [8-11]. IVC type ASDs tend to have more complex anatomy, resulting in incomplete closure compared to other ASDs [9]. Complete diversion of the IVC into the LA would present immediately intraoperatively or postoperatively with severe cyanosis or hypoxia, and misrecognition of the defect can result in death [8-10]. Delayed presentation of hypoxemia many years later could be masked by partial diversion of IVC to the LA, relief of pulmonary congestion, and the extent of IVC stenosis [8-10], which could explain why our patient became symptomatic forty years later.

Our case report demonstrates the importance of utilizing TEE guidance with agitated saline contrast echocardiogram to recognize anatomic variants of the Eustachian valve and treat complications that can arise from misinterpretation. The patient’s chronic hypoxemia was attributed to the right-to-left shunting from the residual ASD that required her to be on supplemental oxygen. The TEE findings confirmed the etiology of the patient’s chronic hypoxemia and significantly impacted the clinical decision to undergo redo sternotomy for redo ASD repair. The potential for the Eustachian valve to be misinterpreted as the inferior rim of the septum is important to be aware of, and TEE can be helpful in evaluating the morphology of Eustachian valve in patients with ASD during surgical correction.

## Conclusions

Accurate identification of the atrial septal rim is critical for correct repair of atrial septal defects. In our case, the eustachian valve was incorrectly used as the inferior rim of the ASD, resulting in significant residual right-to-left shunting. Accurate echocardiography imaging was able to diagnose the iatrogenic IVC type sinus venosus ASD, which led to successful repair of the residual ASD. For patients with atrial right-to-left shunting without pulmonary hypertension, it is important to consider eustachian valves as a possible etiology, and imaging can assist with diagnosis and management. Cardiologists, cardiac anesthesiologists, and cardiothoracic surgeons should be cognizant of this significant, rare complication during initial congenital ASD surgical repair.
